# Beat Gestures for Comprehension and Recall: Differential Effects of Language Learners and Native Listeners

**DOI:** 10.3389/fpsyg.2020.575929

**Published:** 2020-10-19

**Authors:** Patrick Louis Rohrer, Elisabeth Delais-Roussarie, Pilar Prieto

**Affiliations:** ^1^Université de Nantes, UMR 6310, Laboratoire de Linguistique de Nantes (LLING), Nantes, France; ^2^Grup d’Estudis de Prosòdia, Department of Translation and Language Sciences, Pompeu Fabra University, Barcelona, Spain; ^3^Institució Catalana de Recerca i Estudis Avançats, Barcelona, Spain

**Keywords:** gesture, comprehension, recall, beat gestures, non-referential gestures, L1/L2

## Abstract

Previous work has shown how native listeners benefit from observing iconic gestures during speech comprehension tasks of both degraded and non-degraded speech. By contrast, effects of the use of gestures in non-native listener populations are less clear and studies have mostly involved iconic gestures. The current study aims to complement these findings by testing the potential beneficial effects of beat gestures (non-referential gestures which are often used for information- and discourse marking) on language recall and discourse comprehension using a narrative-drawing task carried out by native and non-native listeners. Using a within-subject design, 51 French intermediate learners of English participated in a narrative-drawing task. Each participant was assigned 8 videos to watch, where a native speaker describes the events of a short comic strip. Videos were presented in random order, in four conditions: in Native listening conditions with frequent, naturally-modeled beat gestures, in Native listening conditions without any gesture, in Non-native listening conditions with frequent, naturally-modeled beat gestures, and in Non-native listening conditions without any gesture. Participants watched each video twice and then immediately recreated the comic strip through their own drawings. Participants’ drawings were then evaluated for discourse comprehension (via their ability to convey the main goals of the narrative through their drawings) and recall (via the number of gesturally-marked elements in the narration that were included in their drawings). Results showed that for native listeners, beat gestures had no significant effect on either recall or comprehension. In non-native speech, however, beat gestures led to significantly lower comprehension and recall scores. These results suggest that frequent, naturally-modeled beat gestures in longer discourses may increase cognitive load for language learners, resulting in negative effects on both memory and language understanding. These findings add to the growing body of literature that suggests that gesture benefits are not a “one-size-fits-all” solution, but rather may be contingent on factors such as language proficiency and gesture rate, particularly in that whenever beat gestures are repeatedly used in discourse, they inherently lose their saliency as markers of important information.

## Introduction

Speech is a multimodal act that allows for listeners to make use of both auditory as well as visual cues to make sense of the incoming message. Numerous studies have shown that speech produced with referential gestures^1^ boost both comprehension and recall in the L1 (Riseborough, 1981; Cohen and Otterbein, 1992; among many others), with very few studies showing no effects (e.g., Austin and Sweller, 2014; Dahl and Ludvigsen, 2014). Similarly, positive results have also been found in the L2 (Sueyoshi and Hardison, 2005; Tellier, 2008; Kelly et al., 2009; Macedonia et al., 2011; among many others). A meta-analysis by Hostetter (2011) which analyzed over 60 studies describes six ways in which referential gestures may boost memory, comprehension, and learning: (i) By being better adapted at conveying spatial information than speech, (ii) by giving additional information that is not in speech, (iii) by having positive effects on the speaker’s speech production, (iv) by presenting information that is redundant with speech, affording listeners additional cues to glean meaning, (v) by capturing a listener’s attention, and (vi) by boosting a positive rapport between speaker and listener. Further evidence of these beneficial effects is found in electrophysiological studies on the semantic integration of referential gestures. A handful of studies have found that iconic gestures that are incongruent with their lexical referent in speech produce large N400 s, indicating difficulty in integrating semantic meaning (e.g., Holle and Gunter, 2007; Kelly et al., 2010 among others).

What is less well understood, however, is under which conditions iconic gestures benefit recall and comprehension processes the most. For example, a recent study by Dargue and Sweller (2020) found that *typically* produced iconic gestures aided comprehension of a short narrative over *atypically* produced iconic gestures (e.g., moving one hand upward while pointing to the ceiling with the other hand to represent the character picking up a bucket). Similarly, electrophysiological studies have also determined that N400 effects can be modulated by factors such as speaker style (i.e., using only iconic gestures, compared to producing iconic gestures along with meaningless grooming movements; Obermeier et al., 2015), the temporal affiliation between the iconic gesture and its lexical referent (Obermeier et al., 2011), noise conditions (Drijvers and Özyürek, 2017), or native-language status (Ibáñez et al., 2010; Drijvers and Özyürek, 2018). The current study aims to deepen our knowledge regarding the factor of native-listener status.

Indeed, an important speaker-external factor that seems to strongly regulate the effectiveness of gesture is native-language status. When directly comparing the effect of gestures on recall and comprehension by native and non-native listeners, a different pattern of results emerges depending on L2 proficiency level. Following previous EEG studies with a gesture-congruency paradigm with referential gestures, Ibáñez et al. (2010) found that while high-proficiency learners of German showed similar patterns to native listeners in N400 modulation, low-proficiency learners showed no modulation. The interpretation of these results was such that when speakers are at a lower proficiency, they do not even attempt to integrate information in gesture. Along those same lines, Drijvers and Özyürek (2018) found that iconic gestures in clear speech conditions resulted in larger N400 components in intermediate-level non-native listeners than native listeners, while no N400 modulation was found for non-native listeners in degraded speech. The authors interpret this larger N400 effect in non-native listeners as evidence that they need to focus more strongly on gestures in clear speech to integrate the semantic information. However, when there are no phonological cues available to help with the process, they no longer make use of gestures for semantic integration.

In a recent eye-tracking study, Drijvers et al. (2019a) expanded upon these results. The authors presented native and highly proficient non-native listeners a set of Dutch verbs that were uttered either with or without gesture, and in clear and degraded speech. Immediately following the presentation of each video stimulus, participants were asked to choose which word they heard from four potential candidates. Even though the results showed that both native and non-native speakers benefited from the presence of gesture for the comprehension of Dutch words produced in isolation, they crucially found that both native and non-native listeners showed more accurate answers and faster response times in the gesture condition than in the no gesture condition. While language status did not affect the accuracy of responses, native listeners answered quicker than non-native listeners in the gesture condition with degraded speech. The eye-tracking data showed that in the gesture condition with degraded speech, while all listeners focused more on the face than the gesture, non-native listeners tended to fixate more on gestures than native listeners. Thus authors suggest that non-native listeners cannot make use of visual information from the mouth when auditory cues are unavailable, and thus look for visual information elsewhere. This is unlike native speakers, who can indeed make use of visual cues from the mouth and integrate information both from manual and mouth movements simultaneously. It is this efficiency in integrating multiple channels of information simultaneously that leads the native speakers to respond faster in the cued recall task described above (see also Drijvers et al., 2019b).

To our knowledge, only one study has assessed the benefits of the presence of iconic gestures on recall and comprehension by native and non-native listeners using larger discourses. Dahl and Ludvigsen (2014) directly compared the effects of iconic gestures in both native and non-native listeners in terms of recall and comprehension in a cartoon picture drawing task. 28 native English speaking adolescents and 46 Norwegian adolescents who had been learning English for 7–8 years participated in the study. Each group of participants were divided into two experimental conditions, resulting in a total of 4 experimental groups: Native listener with gesture (NL-G), Native listener without gesture (NL-NonG), Foreign listener with gesture (FL-G), and Foreign listener without gesture (FL-NonG). Each group saw the same 4 picture descriptions presented in English, differing only in whether referential gestures were present or not. Upon watching each video, participants were asked to reproduce the picture that had just been described. Their drawings were evaluated in terms of explicit recall (the presence of elements explicitly described in the discourse), implicit comprehension (the presence of logically implied elements), distortions (elements that were present but inaccurately portrayed), and based on these measures, a composite score was calculated. They found that the native language groups performed similarly on the task regardless of the presence or absence of gesture. In the FL groups, however, the G group showed much higher scores of recall and comprehension, and fewer distortions than their NonG counterparts. Indeed, the FL-G group performed just as well as both NL groups. These results suggest that referential gestures may not have an effect in native listeners, while non-native listeners benefit from information coded in gesture.

Importantly, compared to their referential counterparts, fewer studies have investigated effects on comprehension and recall when there is no lexico-semantic meaning associated with the gesture^2^. Indeed, non-referential beat gestures are one of the most common types of gesture that are produced by speakers, particularly the case in academic contexts where these gestures predominate at rates of up to 94.6% of the gesture types produced (Shattuck-Hufnagel and Ren, 2018, see also Rohrer et al., 2019 for similar results). These gestures (much like their referential counterparts) are also integrated with speech prosody (often co-occurring with pitch accentuation), and their presence can actually modulate a listener’s perception of prominence (see Krahmer and Swerts, 2007; Bosker and Peeters, 2020). Further, non-referential beat gestures have important discursive and pragmatic functions, such as marking information structure (Im and Baumann, 2020), epistemic stance (Prieto et al., 2018; Shattuck-Hufnagel and Prieto, 2019), among others. Indeed, these gestures work with prosodic prominence to act as “highlighters” to important information in speech, potentially increasing listeners’ attention to key words in speech. Thus it seems important to understand how these movements are processed by listeners and can potentially aid in discourse comprehension and recall. This is especially true in the case of non-native listeners, as these movements may aid in determining important aspects of speech and boosting comprehension, particularly in the language classroom. Conversely, they may also be a distraction from concentrating on decoding speech in the auditory domain, due to their non-imagistic nature. To our knowledge no study has assessed the effects of beat gestures on comprehension and recall by non-native listeners. The current study investigates for the first time the potential beneficial effects of beat gestures on language recall and comprehension of a narrative task by both native and non-native listeners.

Recent electrophysiological evidence has helped in obtaining more insight on the integration of non-referential gestures with speech, revealing that non-referential beat gestures boost attention and can help ease semantic integration. An early study by Biau and Soto-Faraco (2013) found that beat gesture-accompanied words elicited a positive shift in the early stages of processing, as well as a later positivity around 200 ms after word onset, showing that gesture is integrated early on in speech processing. Similarly, a study by Dimitrova et al. (2016) found that beat gestures elicited a positivity around 300 ms after word onset. They attribute this to a “novel P3a” component that is said to reflect increased attention. These two studies, when taken together, support the idea of beat gestures working as a “speech highlighter,” boosting attention. Another study by Wang and Chu (2013) showed that beat gestures elicited smaller N400 components, independently of pitch accentuation. Thus, the authors conclude that beat gestures attract attention to focused words, ultimately facilitating their semantic integration. However, while electrophysiological studies seem to suggest that non-referential beat gestures boost attention and ease semantic integration, behavioral studies on these gestures have found conflicting results on their effects on recall and comprehension patterns.

Despite the aforementioned electrophysiological results, behavioral studies have found mixed results when assessing the use of non-referential beat gestures on recall and comprehension patterns, both in adults and children. Comparing gesture types, Feyereisen (2006) exposed adults to 26 sentences, where 10 sentences contained a referential gesture, 10 contained a non-referential gesture, and 6 were filler sentences. A free-recall task showed that the participants remembered the sentences with referential gestures more than those with non-referential gestures. On the other hand, So et al. (2012) found that when presenting lists of single words accompanied by either iconic, beat, or no gesture, adults benefited equally from both iconic and beat gestures, while children only benefited from iconic gesture. However, the previous two studies presented sentences and words without any context. Again looking at both adults and children, Austin and Sweller (2014) investigated the effects of different gesture types on the recall of spatial directions. Participants were shown a Lego base plate with arranged Lego pieces representing different destinations in a town. Participants were then told by the researcher the path the Lego man took. The researcher described the path in one of three conditions: (a) no gesture, (b) producing 20 beat gestures, or (c) producing a combination of gestures (iconic, deictic, metaphoric, and beat gestures, *N* = 5 per type). After hearing the spatial direction describing the Lego man’s path and a 120 s join-the-dots filler activity, participants were asked to recount the path that was described to them. Contrary to the results from So et al. (2012), they found that children did benefit from both “combined” gesture and beat gesture conditions, while adults did not show any beneficial effects from either gesture condition. Further studies with children have shown mixed results. While studies like Igualada et al. (2017) and Llanes-Coromina et al. (2018) found beneficial effects of beat gestures in lists and short discourse contexts with one target beat gesture per sentence, Macoun and Sweller (2016) found that there was no benefit from the presence of beat gesture produced in larger narrations describing a girl’s afternoon in the park with her family. When comparing the effects of non-referential beat gestures, most studies have justified their disparate results by focusing on methodological differences, particularly in terms of stimuli presentation patterns. Some studies presented single words or sentences out of context, while others offered longer narratives of varying sizes. It is important to note that the studies on children seem to suggest that beat gestures are most effective when marking focused information in a pragmatically relevant context. While Igualada et al. (2017) and Llanes-Coromina et al. (2018) used short discourses or lists of words with one gesture occurring in a pragmatically relevant position, studies by Austin and Sweller (2014) and Macoun and Sweller (2016) used a more difficult task with a 2-min narrative with a larger occurrence of beat gestures marking the same words as in the referential gesture condition [20 gestures within 10 target sentences for the Beat gesture condition in Austin and Sweller (2014); 10 gestures within a 2 min narrative for the Beat gesture condition in Macoun and Sweller, 2016]. In this context we think that it is especially relevant to assess the effects of beat gestures in natural speech conditions, which may contain multiple gestures within one narration.

Two studies with adults complemented the data obtained with children, and took into account the relationship between beat gestures and prosody. They showed that gestures are most effective when coupling with prosody to mark contrastively focused information in a pragmatically relevant context. Kushch and Prieto (2016) used larger discourses that contained two contrastive sets within the narrative. The discourses were produced so that prominence could either be given prosodically (through L + H^∗^ pitch accentuation) or prosodically and gesturally (with both a pitch accent and a non-referential beat gesture). These conditions could either appear on the first contrastive pair (where the second pair would be unaccented) or vice versa, resulting in four possible configurations. 20 native Catalan speaking participants watched the discourses and were subsequently given a cloze task, where they had to fill in the words that were contrastively focused from each pair. They found that beat gestures boosted recall significantly more than prosodic prominence alone, and that this effect was even greater when it accompanied the first contrastive pair in discourse. These results were further refined in a more recent study by Morett and Fraundorf (2019). Using similar discourses, they manipulated the conditions to have beat gesture present or absent, and accenting be either presentational (H^∗^) or contrastive (L + H^∗^). While they did not find a main effect of gesture on the recall of information, they did find that contrastively marked information accompanied by a beat gesture was remembered more than presentational information when it was marked with a beat gesture. When gestures were absent, there was no effect of pitch accent type. In other words, beat gestures seem to modulate the efficacy of contrastively marked prominence. Thus, these studies suggest that the gesture’s pragmatic function is also a factor that affects beat gesture’s efficiency in boosting recall and comprehension.

All in all, there is a clear need to assess why non-referential beat gestures seem to have a positive impact on language processing in some instances but not in others. In this regard, following up on recent studies focusing on referential gestures, some research has begun investigating *under which conditions* beat gestures are helpful. To our knowledge, only three studies have assessed the role of beat gestures for non-native listeners, particularly focusing on their effects in novel vocabulary learning, with mixed results. Levantinou and Navarretta (2015) followed the same methodology as So et al. (2012) with presenting individual words with or without iconic gesture, beat gesture, or no gesture. They found that only iconic gestures boosted recall, and that there was no significant difference between the beat and no gesture conditions. The authors claimed that beat gestures may have in fact increased the learners’ cognitive load, as they have not yet learned how to interpret these gestures. Another study by Kushch et al. (2018) presented novel Russian vocabulary words to naïve Catalan learners in a carrier sentence, such as “Bossa es diu ‘sumka’ en rus” (translation: “*Bag is called ‘sumka’ in Russian*”). The target word (*sumka*) was presented in 4 conditions: Accompaniment with neither a (L + H^∗^) pitch accent nor a gesture; Accompanied with a (L + H^∗^) pitch accent (no gesture); Accompanied with a gesture (no pitch accent); or Accompanied with both a (L + H^∗^) pitch accent and gesture. They found that the participants recalled best when target words were produced with both a gesture and a pitch accent. When only one prominence was produced, pitch accented words were better remembered than words produced with beat gesture only. The authors thus claimed that beat gestures can be beneficial in restricted learning contexts and when they co-occur with focal pitch accents. Finally, a study by Morett (2014) used an interactive word teaching and learning task with pairs of native English speakers with no knowledge of Hungarian to assess gesture’s effect on the recall of novel vocabulary. For each pair, one participant was designated as the “explainer” and the other as the “learner.” The explainer had to teach a total of 20 novel Hungarian words. After the presentation of each word, the explainer had to teach the learner the novel vocabulary word “however, they thought [the learner] would learn it best” (i.e., they had no specific instructions regarding gesture production). The entire interaction between the two participants was filmed. After the filmed learning phase, participants had to take a recall test. Gesture’s impact was determined by using multiple regression analysis to examine the relationship between gesture production by both participants during the learning phase and their recall scores. They found that observing gesture did not predict word recall for either participant, regardless of type. However, explainers’ production of beat gestures did predict their own word recall, while learners’ representational gesture production predicted their own word recall. The author explains that these divergent results may be due to the fact that learners may have used representational gesture to enrich the conceptual links between the new words and their referents, while the explainers may have made use of reinforced verbal associations that were established while using beat gestures to convey the meaning of target words. The overall results from this study suggest that gesture production is more beneficial than their mere perception, and in regards to beat gestures, they may be beneficial for different speakers in different contexts. Thus, studies involving the use of beat gesture in L2 have found conflicting results. Further, none of these studies have directly compared native listeners to non-native listeners in the recall and comprehension of complex discourses.

In sum, the previous research on the effects of non-referential beat gestures for recall and comprehension has shown mixed results, where positive results have generally been shown when beat gestures are used in pragmatically restricted contexts, e.g., marking contrastively focused information. Less is known regarding the effects of beat gesture production that has been modeled after natural discursive speech, reflecting more natural, real-world experiences that listeners encounter (yet see Austin and Sweller, 2014; Macoun and Sweller, 2016). Thus it seems important to see the effects of these gestures in more natural speech conditions, which may contain multiple gestures within one narration. Importantly, no study with beat gestures has directly compared between native and non-native listeners. Thus the main aim of the study is to compare the effects of beat gestures between native and low-intermediate-level non-native listeners in a narrative-drawing task. This population was chosen as some studies have suggested that gestures may be more beneficial for lower-level learners (see Sueyoshi and Hardison, 2005; Morett, 2014). We believe that non-referential beat gestures may help non-native listeners as they index key words in the narrative, potentially boosting their attention to these aspects and consequently aiding in their recall. Further, as mentioned before, beat gestures serve discourse and information structure marking functions, which may boost discourse comprehension in terms of understanding the relationship between the elements and actions in the narrative. However, it is quite possible that compared to native speakers, these more complex, naturalistic conditions may lead to cognitive overload (i.e., processing costs beyond the listener’s cognitive capacity) for low-intermediate-level non-native listeners with too much visual stimulation, causing them to focus on the movements and miss out on important information being presented orally (e.g., Drijvers et al., 2019a). Following Dahl and Ludvigsen (2014), a narrative-drawing task was chosen as it offers a blank slate to determine what information is recalled and understood from the narrative, without the implications of using comprehension questions which may assess recall and comprehension in a more precise manner but require language processing and production skills to answer. This is particularly relevant for low-intermediate L2 learners. The current study will thus give insight on the effects of non-referential gestures on recall and discourse comprehension in more natural contexts and particularly by low-intermediate non-native listeners, which could potentially guide our understanding on not just *if* these gestures are beneficial, but *when* they are beneficial. The results may also eventually be applied in language learning contexts, where gestures may be used to potentially boost vocabulary learning or facilitate oral comprehension in the L2.

## Materials and Methods

### Participants

A total of 51 participants (41 females, 9 males, and 1 non-binary, *M*_age_ = 23.28, SD = 7.2) were recruited from 4 intermediate-level English classes at the University of Nantes. One of the English classes where participants were recruited from was for second year undergraduate students studying English as part of their Language Sciences degree (*N* = 15). The other three English classes were offered by the *Service Universitaire des Langues* (SUL) at the University of Nantes (*N* = 13, 13, and 10, respectively). These courses are open to all students and faculty wishing to improve their English level. Professors from each course agreed to dedicate one class session to the experiment. All participants gave informed consent.

In order to assess L2 level, participants in the 3 SUL classes had taken the University of Grenoble’s SELF language assessment^3^ test before enrolling in the class. Students who did not have a SELF score were given the 20-min General English Test offered by International House London^4^ before the task. A large majority of participants reported an intermediate level of English (CEFR: A1 = 5%, A2 = 5%, B1 = 35%, B2 = 50%, and C1 = 5%).

Eight students were removed from analyses. First, 6 students were removed because they reported languages other than French as their L1. Two students were removed from analyses for having a C1 level in English. Since previous research has shown that advanced learners attend to gestures in much the same way as native listeners (Ibáñez et al., 2010), these participants’ profiles were deemed too native-like and did not match the profile of the rest of the students.

### Materials

#### Stimuli Creation for the Drawing Task

**FIGURE 1:**
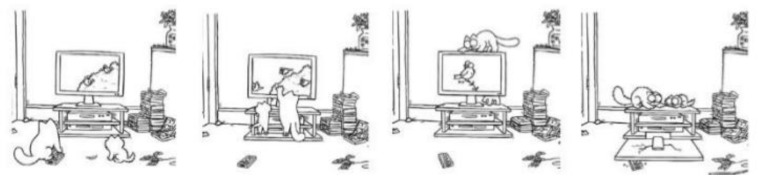
An example comic strip, taken from “Simon’s Cat” by Simon Tofield. Reprinted with permission. © Simon’s Cat Ltd.

A subset of 8 comic strip illustrations was chosen from the Simon’s Cat comic series that were used in Cravotta et al. (2019). These 8 comic strips were chosen based on the ease of translating the illustrations to a short narrative that could be understood by low-intermediate level language learners. A short narration was then written for each comic in both French and English. All narratives followed the same basic structure where each square in the comic trip was introduced by a sequencing marker (“First,” “Next,” “Then,” and “Finally”) which described the development of the narrative, followed by a short description of the orientation of items in the square or actions that have occurred since the previous square. See Figure 1 for an example comic strip; its corresponding narration can be found in section “Appendix A.”

Gesture placement for the final stimuli was then determined by recordings of two native speakers in each language who read the narrations aloud. The 4 speakers had no knowledge of the purpose of the study and were merely asked to read the narration while being “expressive with their hands.” In doing so, it was possible to determine the most natural lexical affiliates in the narrative to be marked with a gesture. A majority of the gestures produced were non-referential in nature. The lexical affiliates of each gesture (regardless of referentiality) produced by each participant for each comic was then determined, and the inclusion of these “gesturally-marked elements” in the final stimuli were determined by three factors. First, a gesturally-marked element was included if at least 3 speakers marked that same word-referent (across languages) was automatically included in the final stimuli. A second factor was gesture salience (i.e., the perception of a large gesture movement or more emphatic gesture). If one of the speakers made a particularly salient gesture on a word (and perhaps one other person also marked that same word with a gesture), then it was included in the final stimuli. The third and final factor was the pertinence of the gesture to the narrative. In other words, if 2 speakers marked a word that contrasted with another element, it was seen as being pertinent to the narrative as it disambiguated two items, and this gesture would be included in the final stimuli. After analyzing the natural speech productions, scripts were created for each narrative that contained the gesturally-marked lexical affiliates in bold for filming. Table 1 shows the average number of gestures per sentence, the total number of gestures, and the duration of each video.

**TABLE 1:** The average number of gestures per sentence, and total number of gestures per comic narrations.

**Comic language**	**English**			**French**		
**Comic number**	**Average N of gestures per sentence**	**Total N gestures**	**Video duration (in seconds)**	**Average N of gestures per sentence**	**Total N gestures**	**Video duration (in seconds)**
1	2.75	22	64 s	2.33	21	64 s
2	1.71	24	84 s	1.92	25	75 s
3	3	21	53 s	3	21	50 s
4	2.08	27	73 s	1.75	21	62 s
5	3	30	77 s	2.91	32	73 s
6	3.09	34	65 s	2.82	31	59 s
7	2.88	23	54 s	2.3	23	53 s
8	1.91	21	61 s	2.1	21	60 s

#### Video Filming, Editing and Validation of the Target Narrations for the Drawing Task

Two female native speakers were recruited to record the spoken narrations in their respective native language. Recordings took place in a professional recording studio at Universitat Pompeu Fabra in Barcelona, and the speakers were paid 10 euros per hour. The actresses were briefly shown the types of gestures they would be making (i.e., beat gesture) and that they would produce them on target words. They were then given opportunities to practice producing the narratives with gestures. While the speakers were relatively free to produce the non-referential gestures as they saw fit, they were given feedback to have a more relaxed, natural style. This was done to avoid disparate differences in gesture salience between the two speakers. In other words, both speakers were trained to produce the gestures in a relaxed and natural way, with most gestures being small up-and-down movements or flips. Each actress then recorded multiple trials of each narration in both the Gesture (G) and the No-Gesture (NG) conditions following a teleprompter which displayed a script of each narrative, with target words to contain a gesture (in the G condition) being marked in capital letters. In order to maintain a natural style, no instructions were given in terms of prosodic emphasis.

**FIGURE 2:**
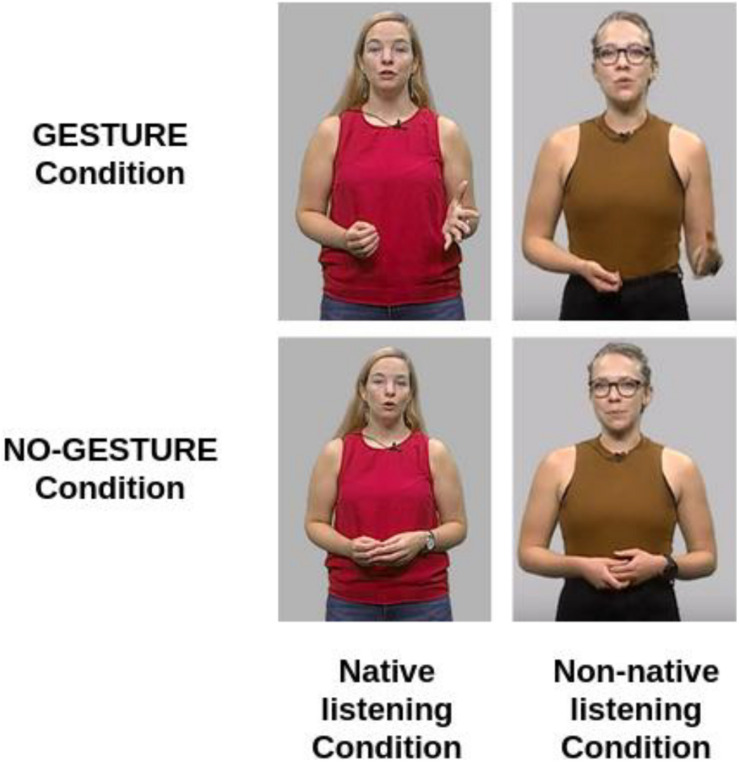
Still-frames taken from the stimuli videos of one narration in each condition.

Following the recording session, videos were then edited in Adobe Premiere Pro (CS6). Videos were edited to show the actresses placed in front of a simple gray background. They were shown from the waist up so that both hands were visible, as well as the face. Again, this was done to keep the stimuli close to real-world situations as possible. The average duration of the edited videos of the target narrations was 63.94 s (±9.27 s) and each narration contained an average of 25 (±5) gestures. Figure 2 shows four still-frames taken from one of the narrations showing each speaker either in the gesture condition, or the no-gesture condition.

To ensure the naturalness of the gestures in the video stimuli, 5 native English speakers, and 6 native French speakers evaluated how natural the gestures seemed for each video of their corresponding language. Each rater evaluated the videos on a Likert scale from 1 to 7, where 7 was the most natural. The French videos received an average score of 4.71 (SD = 1.58) while the English videos received an average score of 5.53 (SD = 1.48). This suggests that raters generally felt that the videos were relatively natural-looking.

#### Stimuli Organization of the Narrations for the Drawing Task

The aforementioned steps resulted in a total of 32 videos, where each narration was video-recorded in four conditions: in native listening conditions with gesture, in native listening conditions without gesture, in non-native listening conditions with gesture, and in non-native listening conditions without gesture. In order to ensure that participants see all of the narratives in different language and gesture conditions in a balanced manner, a Latin-square method allowed for the division of the stimuli into 4 stimuli lists (see Table 2), where narrations were balanced for the language listening condition and gesture condition in each list. In other words, each list contained the 8 narrations, but the lists differed in terms of the language and gesture conditions that were presented for each narration. By organizing the stimuli this way, it was possible to avoid any bias stemming from the individual narrations themselves. Each stimuli list was then uploaded to SurveyGizmo as an individual online survey for the presentation of the stimuli. Each survey followed the same structure. An initial screen gave instructions. The survey then alternated between video presentation screens and screens that instructed participants to draw. The survey ended with a “Thank You” screen that informed participants that the experiment had ended (see section “Drawing task” for more details).

**TABLE 2:** The distribution of language and gesture conditions by comic narration into four counter-balanced lists.

**Comic narration number**	**List 1**	**List 2**	**List 3**	**List 4**
1	NON-NATIVE-G	NATIVE-NG	NATIVE-G	NON-NATIVE-NG
2	NON-NATIVE-NG	NON-NATIVE-G	NATIVE-NG	NATIVE-G
3	NATIVE-G	NON-NATIVE-NG	NON-NATIVE-G	NATIVE-NG
4	NATIVE-NG	NATIVE-G	NON-NATIVE-NG	NON-NATIVE-G
5	NON-NATIVE-G	NATIVE-NG	NATIVE-G	NON-NATIVE-NG
6	NON-NATIVE-NG	NON-NATIVE-G	NATIVE.-NG	NATIVE-G
7	NATIVE-G	NON-NATIVE-NG	NON-NATIVE-G	NATIVE-NG
8	NATIVE-NG	NATIVE-G	NON-NATIVE-NG	NON-NATIVE-G

### Procedure

An online linguistic background survey was emailed to each participant to be completed before the drawing task in order to collect each participant’s personal information (e.g., gender, age, level of study), as well as to assess their L1. Participants were also asked to bring their own laptops and headphones on the day of the drawing task, which would allow them to access the survey online. Immediately before the session, each participant was given a link to their corresponding list’s online survey containing the stimuli videos.

#### Drawing Task

The drawing task was carried out in 4 English classes containing about 15 students each. The participants did the task individually and in a self-paced manner. It was carried out in a quiet classroom under the supervision of the class instructor^5^. Each participant was given a small task booklet that contained an instructions page, followed by a set of 8 pages, where each page contained 6 large squares for the participants to draw their interpretations of the comic narrations. Then, participants were informed that they were going to perform a narrative comprehension task, and were directed to read the instructions carefully. Instructions (adapted from Dahl and Ludvigsen, 2014) were available in both French and English and were as follows:

You are about to watch 8 short video clips, half of them in French, and half of them in English. Each clip is a description of a different humorous comic strip. Watch to the first description and create a picture in your mind of what this comic strip looks like. Try to remember as many details as possible. You are not allowed to draw while you are watching the video. Once the video has ended, try to draw the comic strip that you just heard described.

The quality of your drawing skills is not the most important thing. What is important is how much you remember of the comic strip that was described and that you show that through what you draw. Try to include as much as possible in the drawing. In case something is hard for you to draw or some element in the drawing seems unclear in the picture, you can write and draw arrows next to the element to clarify what it is.

You are given a page with 6 squares to draw in. Note that you can use as many or as few of the squares as you think are appropriate for the story. That is, if you think the comic being describing is only 3 squares long, you draw the entire comic in three squares. Try to use all of the space within each square.

Once you have finished the drawing, you may move on to the next video description.

Upon reading the instructions, participants were directed to access the survey via the link that they had received by e-mail. The online survey again gave a more concise version of the above instructions and once the participant acknowledged they understood and were prepared, they began the stimuli presentation. Stimuli from the participants’ assigned list were presented in random order, and the presentation screen contained an embedded video. This screen remained accessible for at least 2 min and 30 s, just enough time to watch each video two times, while not allowing participants to watch a third time. After watching the video two times (or when the time limit was reached), the survey would proceed to a screen that instructed the students to draw the comic that had just been described in the video. There was no time limit on this screen, so participants could take the time necessary to complete their drawing. Once completed with their drawing, the participant then proceeded to the next random video stimulus. Upon completing the survey and all of the drawings for the 8 narrations, students turned their booklet into the instructor.

#### Scoring of the Drawing Task

To evaluate **explicit recall**, a list of all the items that were gesturally marked in the gesture condition was created for each narration (see Table 1 for the number of gesturally marked items per narration). These lists served as checklists when determining whether these specific items were accurately remembered or not. The main author carried out all of the scoring while unaware of which condition the drawing pertained to. For each item in the checklist of a given comic description, if the element is clearly remembered and present in the drawing, a score of 2 is given. If the element was not remembered exactly as described or it is ambiguous whether the element was remembered clearly or not, a score of 1 was given. This score was used for cases in which memory of the element was distorted. When the element is not present at all in the drawing, a score of 0 was given. For example, if the narration had the sentence “The cat is sleeping on a **rectangular** rug” (bold indicates the lexical affiliate of the gesture in the G-condition), and the drawing shows a rectangular rug, the participant received 2 points. If the drawing shows a circular rug, the participant would receive only 1 point. If there is no rug in the drawing, the participant received 0 points. The maximum number of points a participant could receive per drawing ranged from 38 to 60 points depending on the number of gesturally marked items in the corresponding narration. While most gesturally marked elements were nouns, verbs, or adjectives that marked focus (e.g., “a **rectangular** rug” or “the cat **jumped** in the air.”), discourse markers “First,” “Next,” “Then,” and “Finally” were also gesturally marked elements. As such, not only were participants’ recall evaluated in terms of remembering particular items or actions, but also in terms of the sequencing of events. See section “Appendix B” for an example scoring of recall for one comic square.

Unlike the current study that uses narratives, the study by Dahl and Ludvigsen (2014) used picture descriptions as stimuli for their student to draw, and they not only looked at explicit recall, but also “implicit comprehension.” They describe implicit comprehension as the participant’s understanding of information that was not explicitly stated in the picture description they heard. For example, they describe the explicit recall and implicit comprehension evaluated in one of their comics, saying: “the placement of a bench was explicitly mentioned in relation to where a dog is in the image… the dog’s placement is explicitly described in relation to a woman whereas the location of the woman in relation to the bench is logically implied via her relationship to the dog.” (p. 820). In order to go beyond investigating explicit recall of specific items that were mentioned in the narratives of the current study, it was decided to also assess their discourse comprehension in terms of the semantic relationship between the different elements (i.e., the narrative’s event structure, see Li et al., 2017). This is distinguished from recall in that while recall tests participants’ ability to retrieve lexical information regarding elements in the story (e.g., the presence of a cat, a television, and 3 birds in the narrative), discourse comprehension measures participant’s understanding of the relationship between these items (e.g., that the cat is **trying to catch** the three birds **that are being televised on the screen**, which ultimately leads to the cat **breaking** the television). As such, each drawing was evaluated on a Likert scale for the general comprehension of the event structure of the narrative. The Likert scale was on a scale of zero to five, where 0 corresponded to absolutely no correspondence between the drawings and the narrative, to 5 indicating a complete understanding of the event structure of the story (see Table 3). See section “Appendix C” for an example scoring of discourse comprehension. Thus each drawing was given a recall score for each gesturally marked element in the narrative, and one single score for discourse comprehension.

**TABLE 3:** The scoring rubric to evaluate comprehension.

**Score**	**Interpretation**	**Description**
0	Not-evaluable	The drawing had no correspondence with any aspect of the narrative or was left blank
1	No understanding of the narrative	Perhaps drew a character or object, but no story development is present
2	Minimal understanding of the narrative	Drew at least one event from the narrative, but minimal story development
3	Partial understanding of the narrative	Drew multiple events from the narrative, understands at least partially the “main goal” but misunderstands some other aspects of the narrative
4	Near complete understanding of the narrative	Clearly understood main goal of the narrative, as well as possibly some other minor details that are implicated in the story
5	Complete understanding of the narrative	Clearly understood the main goal of the narrative, as well as other minor details that are implicated in the story

#### Reliability

Interrater reliability was calculated using Fleiss’ kappa with three additional raters evaluating both recall and comprehension for a total of 64 drawings, representing 18.6% of all the data. The calculation of recall scores were based on evaluators’ individual scores for each gesturally marked item (where a score of 2 indicates perfect recall, a score of 1 indicates distorted recall or ambiguity, and a score of 0 indicates no recall, see section “Scoring of the drawing task”). Fleiss’ kappa showed that there was good agreement between the raters’ scores, κ = 0.713 (95% CI, 0.713 to 0.714, *p* < 0.001).

In terms of comprehension, reliability was calculated using the individual comprehension scores. Fleiss’ kappa showed moderate agreement between the raters, κ = 0.529 (95% CI, 0.527 to 0.531, *p* < 0.001). Reliability was further calculated by grouping the individual comprehension scores so that a score of 1 or 2 would be binned as “low comprehension” and a score of 4 or 5 would be binned as “high comprehension.” Fleiss’ kappa showed good agreement between the raters, κ = 0.723 (95% CI, 0.720 to 0.725, *p* < 0.001).

### Statistical Analyses

Two Generalized Linear Mixed Models (GLMMs) were applied to the recall and comprehension scores using the *glmmTMB* package in R (Brooks et al., 2017). For both GLMMs, the fixed factors were Condition (two levels: Gesture and No Gesture), Language (two levels: Native and Non-native) as well as their interaction. To determine the random effects structure for each GLMM, a series of Linear Mixed Models were modeled using all the potential combinations of random effects, from the most complex structure to a basic model containing no random effects. Structures that did not produce any converge problems were then compared using the “compare performance” function from the *performance* package (Lüdecke and Makowski, 2019) to identify the best fitting model for the data. In other words, this process assesses all of the possible random effects structures and returns the best-fitting model. For both dependent variables, the best fitting model was a random effects structure which included a random intercept for item (i.e., the individual comic narrative) and a random slope for Language by Participant. Omnibus test results are described below, as well as the results from a series of Bonferroni pairwise tests carried out with the *emmeans* package (Lenth, 2019), which includes a measure of effect size (via Cohen’s d).

## Results

**FIGURE 3:**
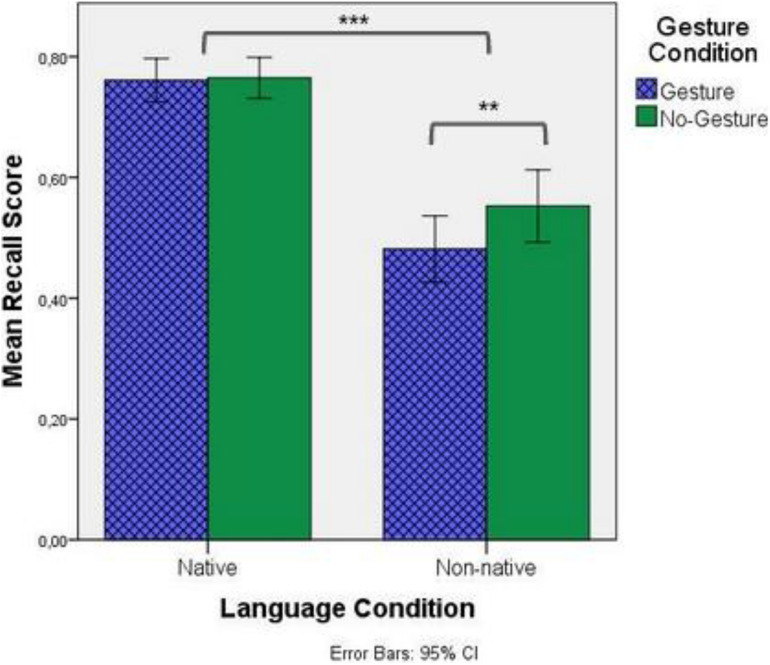
Mean recall scores by Language and Gesture conditions. “**” Refers to a *p*-value less than 0.01, while “***” refers to a *p*-value less than 0.001.

Figure 3 below shows the average recall score (in%) for both Language and Gesture Conditions. Results of the GLMM with recall score as the dependent variable reveal a significant main effects of Language [χ2(1) = 88.297, *p* < 0.001] and Condition [χ2(1) = 5.248, *p* = 0.022], as well as a significant interaction between Language and Condition [χ2(1) = 4.150, *p* = 0.042]. *Post hoc* comparisons showed that participants did significantly better in Native listening conditions than in Non-native listening conditions (*d* = −1.83, *p* < 0.001) and did significantly better in the No-Gesture condition than the Gesture condition (*d* = −0.25, *p* = 0.023). As for the significant interaction, while gesture had no impact on recall in Native listening conditions (*d* = −0.03, *p* = 0.855), participants scored significantly better in the No-Gesture condition than in the Gesture condition when in Non-native listening conditions (*d* = −0.47, *p* = 0.002). From these results, it seems that while beat gesture has no major effect for native listeners, they negatively impact recall when participants listen to a non-native language.

**FIGURE 4:**
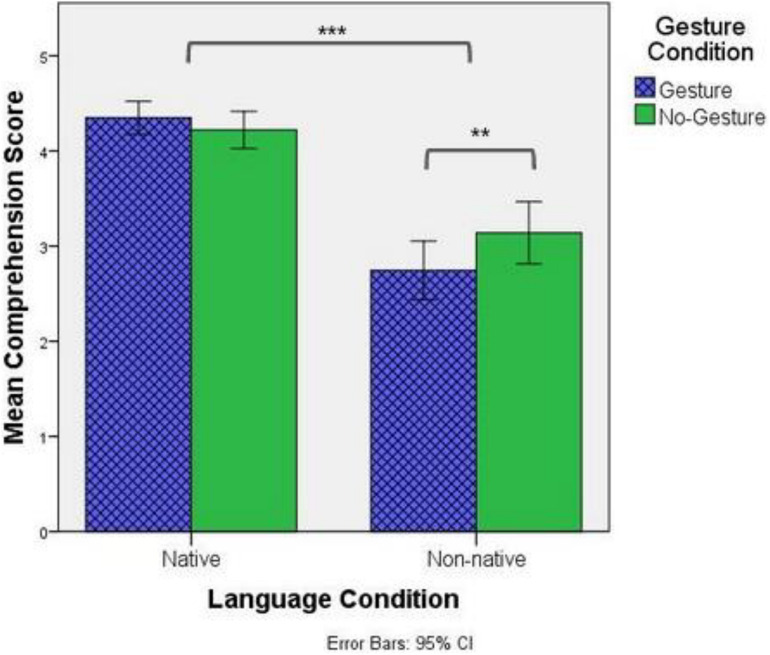
Mean comprehension scores by Language and Gesture condition. “**” Refers to a *p*-value less than 0.01, while “***” refers to a *p*-value less than 0.001.

Figure 4 below shows the average comprehension score for both Language and Gesture Conditions. Results of the GLMM with comprehension score as the dependent variable reveal a significant main effects of Language [χ2(1) = 68.398, *p* < 0.001] and a significant interaction between Language and Condition [χ2(1) = 9.673, *p* = 0.002]. Similar to the recall scores, *post hoc* comparisons showed that participants did significantly better in Native listening conditions than in Non-native listening conditions [*d* = −1.84, *p* < 0.001]. In regards to the interaction, while gesture had no impact on comprehension in Native listening conditions [*d* = 0.18, *p* = 0.249], participants scored significantly better in the No-Gesture condition than in the Gesture condition when in Non-native listening conditions [*d* = −0.49, *p* = 0.001]. Thus similar to the results on recall, it seems that while beat gesture has no major effect on comprehension for native listeners, they negatively impact comprehension when participants listen to a non-native language.

When comparing the recall and comprehension scores regardless of condition, we find a significant, positive correlation between the two scores [*r*(342) = 0.893, *p* < 0.001], suggesting that as participants remembered more individual items in the narratives, they also better understood the overall event structure of the narrative.

## Discussion

The results of the present investigation show that while the presence or absence of beat gestures in discourse does not affect either recall or comprehension of complex narrative speech for native listeners, when those same listeners are exposed to speech that is not in their native language and of which they have an intermediate proficiency level, non-referential beat gestures significantly impede both recall and comprehension.

First, the results in terms of the non-beneficial effects of non-referential beat gesture on native language contexts contribute to expand and refine our knowledge about the benefits of gesture in recall and comprehension processes and further understand some of the reasons behind the conflicting results. Our results are in line with results from the studies by Dahl and Ludvigsen (2014) and Austin and Sweller (2014), where neither study found any benefit of gestures (referential gestures in the case of the former, neither referential nor non-referential in the case of the latter) for information recall. Importantly these results contrast with other studies that report positive results for both of these gestures. By looking closely at the stimuli of the two studies it is particularly interesting to note that methodologically these reflect the methodology in the current study in terms of the stimuli used. Particularly regarding the length of the narratives and the number of non-referential gestures used, the current study as well as both Austin and Sweller’s (2014) and Dahl and Ludvigsen’s (2014) studies were similar. Interestingly, the stimuli were substantially longer and contained more gestures than studies that found positive effects (e.g., Kushch and Prieto, 2016). Thus a potential reason that these gestures do not boost recall and comprehension is gesture rate, i.e., the fact that speakers repeatedly used gestures (in our study, between two to three lexical items were marked with a gesture per sentence, see Table 1).

Thus our interpretation of the non-beneficial effects of non-referential beat gestures in the native speaker group is that having a high rate of gesture may have “bleached” their pragmatic intent, provoking changes in the listener’s processing of discourse. By contrast, previous evidence has shown that when non-referential beat gestures occur with the specific pragmatic function of contrastive focus (e.g., Wang and Chu, 2013; Dimitrova et al., 2016; Kushch and Prieto, 2016; Llanes-Coromina et al., 2018; Morett and Fraundorf, 2019) or highlighting one of the items in a list (Igualada et al., 2017), these gestures are beneficial for recall or comprehension. In the current study, the speakers after which the target stimuli were modeled were instructed to “speak expressively with their hands” which may have ultimately led to an exaggerated performance in terms of the number of non-referential beat gestures that were produced. This increase in the number of gestures may have hidden any real pragmatic relevance to them, ultimately using non-referential beats that were no longer pragmatically relevant. Most of the non-referential beat gestures that were produced in our target narrations marked information structure (i.e., new referents, broad focus, narrow focus, etc.). That is, they marked information that the speaker would have deemed “important.” However, it might well be that in marking too many elements as important in discourse, the inherent property of marking something as separate (i.e., “important”) is reduced, ultimately reducing the effectiveness of non-referential beat gestures as highlighters of important information (McNeill, 1992; see also Biau and Soto-Faraco, 2013; Dimitrova et al., 2016, among others). This is also in direct contrast with studies that showed benefits in semantic integration and comprehension (e.g., Wang and Chu, 2013; Llanes-Coromina et al., 2018), where the presence of a beat gesture on a contrastively marked element may have increased the listener’s interpretation of speaker certainty, reducing doubt in their interpretation of speech and ultimately aiding in semantic processing. As the current study did not use gestures to merely mark contrastive elements, they may not have had this effect of reducing the certainty of the listener’s semantic interpretation.

Parallels of what we can classify as a *gesture rate effect* can be drawn from the interpretation of typographic prominence (e.g., capital letters). Scott and Jackson (2020) describe how using capitalized letters in the written modality can give the reader an impression of emphasis. However, a stylistic choice to write entirely in capital letters causes the reader to no longer interpret capitalization as a marker of emphasis and thus must do something different to mark emphasis (e.g., putting an emphasized element in italics). Thus, it is sensible to conclude that whenever beat gestures are repeatedly used in discourse, they inherently lose their saliency as markers of important information.

Moreover, presumably the fact that repeatedly used beat gestures triggered not only a loss of their pragmatic saliency but also potentially led our listeners to adapt their reliance on gesture based on speaker style. Indeed, two studies have already shown how listeners adapt to the gestural behavior of their interlocutor. The previously mentioned EEG study by Obermeier et al. (2015) showed that when listeners see speakers producing both meaningless grooming gestures along with iconic gestures, they do not process their iconic gestures as strongly as when speakers did not perform any grooming gestures. Similarly, a recent behavioral study with beat gestures by Morett and Fraundorf (2019) defended a top-down approach in discourse processing. This “top-down” approach implies that listeners attune to the gestural habits of speakers and make inferences about their intentions based on their behavior (as opposed to a bottom-up approach where merely the presence of cues in the speech signal guide the listener’s interpretation). Within the interpretation of these studies, it seems as though the native-listeners were exposed to repeatedly produced beat gestures, making these gestures unreliable and ultimately failing to raise attention to important information in speech and reducing any potential benefit for recall.

Second, along with recent studies on referential gestures, the results of the present investigation showed that beats had negative effects for low-intermediate language learners. Our results complement and expand previous findings showing that lower-level language learners show increased processing cost when gestures are present and that gesture processing stops when speech becomes too difficult to understand (Ibáñez et al., 2010; Drijvers and Özyürek, 2018). In terms of our results, participants may have been at a disadvantage from increased processing costs for gesture, doubled with the lack of semantic information to be gleaned from these movements. As such, perhaps the non-native listeners at a low-intermediate level are still dependent on clear semantic meaning in gestures. By contrast, the studies by Dahl and Ludvigsen (2014) and Drijvers et al. (2019a), who found positive effects of iconic gestures on recall and comprehension processes, recruited advanced learners and exposed them to referential gestures, whereas in the current study, the non-native listeners had a low-intermediate level and were exposed to non-referential gestures.

Our study is not the first to find negative effects for gestures. In terms of L2 novel word learning, Kelly and Lee (2012) found that when teaching word pairs that differ by only a geminate, the presence of referential gestures had a negative effect on the participants’ word learning. However, the gestures were indeed beneficial whenever the word pair differed by both a geminate and their segmental composition. The authors thus suggest that gestures are only helpful when phonetic demands are low. Another study using an electrophysiological paradigm by Zhang et al. (2020) used naturalistic stimuli to investigate how multimodal cues interact in discourse processing, notably the N400. This study particularly stands out, as they used natural stimuli that contained multiple gestures (and often beat gestures). Interestingly, they found that when controlling for linguistic surprisal for each word, referential gestures had a tendency to lower the N400 (generally interpreted as easing semantic integration), while beat gestures tended to have the opposite effect.

The findings from the current study are limited in a few aspects. First, the actresses that were featured in the stimuli were given no specific instructions in terms of prosody in order to maintain the naturalness of the stimuli (i.e., to avoid having to overlay audio tracks and blur faces, etc.). While beat gestures tend to associate with speech prominence, studies have shown that the production of a beat gesture affects how acoustic prominence is realized in speech (e.g., Krahmer and Swerts, 2007; Pouw et al., 2020). Thus it is possible that differences in the phonetic realization of prominence may have had an effect. Conversely, other studies have also shown that when prosody is held constant, the presence of a beat gesture boosts the perception of speech prominence (Krahmer and Swerts, 2007; Bosker and Peeters, 2020). Even though our materials were controlled for the presence of pitch accentuation in beat positions across conditions, the fact that speech production was not kept completely constant does not rule out the possibility that pitch range differences might have had an effect on the results. Thus, future studies should control for phonetic differences in prosodic prominences to flush out to what extent it is modulation in the visual or auditory cues to prominence that are the driving factor behind these effects.

Another limitation of this study regards the methodological choices. The study only looked at intermediate learners of English. By adding high proficient learners, it would have been possible to flush out any proficiency-level effects. This could potentially show at what stage in learning non-referential gestures stop being detrimental for recall and comprehension in language learners. Another limitation is in regards to the processing costs of our participants. Also, by adding an electrophysiological element to the study, we would have been able to directly measure these processing costs. The task itself may have been a limiting factor, particularly for participants who did not feel confident in drawing. Though participants were reassured by the experimenter that their drawings could be simple stick figures and that they could write words and draw arrows for things that may have been difficult to draw, and all of them expressed enough confidence in an informal way, it would be good for future studies to take a measure of drawing confidence in the task and factor this variable into the statistical modeling.

Finally, it is important also to consider that all of the participants in the current study were native French learners of English. As such, we cannot discard the possibility of L1 language effects in the results of the effects of beats in the L2. While non-referential beat gestures show similar patterns of integration with speech prominence in both languages (see Shattuck-Hufnagel and Ren, 2018; Rohrer et al., 2019), in terms of focus marking, French makes use of thematic structures more often than prosodic focus, and non-referential beat gestures tend to align more with the prosodic focusing than thematic structures (Ferré, 2011). In other words, native French listeners may rely less on prosodic and gestural marking for focus. English, on the other hand does not use clefting strategies as often to mark focus (e.g., Vander Klok et al., 2018), potentially making beat gestures a more reliable marker of focus than in French. As such, it would be interesting to see if similar results were found with native English learners of French, or in a completely different pair of languages. In the case that there is no difference between populations, inherent language differences could be ruled out.

All in all, the current study adds to our understanding of the role of gesture in recall and comprehension processes by giving insight into *when* gestures are beneficial for listeners, both native and non-native. Methodologically, the results of our study highlight the need for researchers to take task complexity into account when interpreting results on gesture-speech integration processes, and particularly the effects the length of the discourse, the pragmatic functions of gesture, and the gesture rate. This is particularly true in second language contexts. While previous positive results could have led language instructors to believe that adding non-referential beat gestures to their discourse would be beneficial for their students, results from the current study suggest that this is not necessarily the case and that degree of proficiency and task complexities are important factors that need to be taken into account. Instructors are encouraged to reflect more on using beat gestures in specific, relevant contexts and to select precisely what information is important for the listener, and finally take into account that level of proficiency in the foreign language is a crucial factor in the processing of gesture-speech integration.

## Data Availability Statement

The raw data supporting the conclusions of this article will be made available by the authors, without undue reservation.

## Ethics Statement

Ethical review and approval was not required for the study on human participants in accordance with the local legislation and institutional requirements. The patients/participants provided their written informed consent to participate in this study. Written informed consent was obtained from the individual(s) for the publication of any potentially identifiable images or data included in this article.

## Author Contributions

PR, ED-R, and PP contributed equally to the development of the research questions, the experimental design, and the discussion of the results. PR carried out the data collection and analysis, and was in charge of the writing of the article, with feedback from ED-R and PP. All authors contributed to the article and approved the submitted version.

## Conflict of Interest

The authors declare that the research was conducted in the absence of any commercial or financial relationships that could be construed as a potential conflict of interest.

## Footnotes

1 Studies on co-speech gestures have widely adopted McNeill’s (1992) classification of gestures as iconic, metaphoric, deictic, or beat gestures. The former three are considered referential in nature, as they make direct references to semantic content in speech.

2 i.e., McNeill’s “beat” gesture, often said to be non-referential because there is no clear semantic reference in speech. Indeed, [McNeill (1992):15] describes these movements as simple up-and-down movements or flicks of the hand or fingers, that seem to be beating to the rhythm of speech. Recently, they have been claimed to have more pragmatic functions. For example, these gestures tend to mark new or contrastive information, or discourse structure (see also Prieto et al., 2018; Shattuck-Hufnagel and Prieto, 2019).

3 Innovalangues: SELF, http://innovalangues.fr/realisations/systeme-d-evaluation-en-langues-a-visee-formative/

4 International House London, https://www.testmylevel.com/

5 As three of the four classes took place at the same time, the first author was present for two of the four experimental sessions. In the two experimental sessions in which the main author was not present, participants were under the supervision of the course instructor who had been debriefed about the details of the procedure. Neither instructor indicated any difficulty or issues while running the experimental session.

## Appendix A: Narration for the Comic in Figure 1 in English and French (Bold, Capitalized Words Indicate a Gesturally Marked Element)

**FIRST**, we see a **LARGE** television on a **TABLE**. On the **TELEVISION** screen, we see **TWO** birds. To the **RIGHT** of the television there are two **STACKS** of magazines on the **FLOOR**, and a **SHELF** with a **FLOWER** vase. A **CAT** and a **SMALL** kitten are sitting in **FRONT** of the **TV**, watching the two birds. The cat, which is on the **LEFT**, has a remote control under his **RIGHT** hand. **NEXT**, we see that the television screen shows **THREE** birds, and both the **CAT** and the **KITTEN** have climbed **ONTO** the table. The **LARGER** cat, now on the **RIGHT**, is reaching his hand toward the screen. **THEN**, we see the **TV** screen shows **ONE** bird on a **BRANCH**. The large cat has climbed on **TOP** of the **TV** screen and is looking **DOWN** at the bird, while the kitten is **UNDER** the large cat, **BEHIND** the television. **FINALLY**, we see the **TWO** cats on the table, looking **SHOCKED**. The **TV** has **FALLEN** to the ground and the back of the television is **CRACKED**.

**D’ABORD**, on voit un **GRAND** téléviseur sur un **MEUBLE**. Sur **L’ECRAN** de la télé, on voit **DEUX** oiseaux. A **DROITE**, il y a **DEUX** piles de magazines et une **ÉTAGÈRE** avec un vase de **FLEURS** dessus. Il y a un **GROS** chat et un **PETIT** chat assis **DEVANT** la télé, en train de regarder les deux oiseaux. Le gros chat, à **GAUCHE**, tient la télécommande dans sa main **DROITE**. **ENSUITE**, on voit sur l’écran **TROIS** oiseaux, et les **DEUX** chats sont montés sur le **MEUBLE**. Le **GROS** chat, maintenant à **DROITE**, lève la main vers l’écran. **PUIS**, on voit sur l’écran **UN** oiseau sur une **BRANCHE**. Le gros chat est **MONTÉ** sur la télé et regarde en **BAS** vers l’oiseau, alors que le petit chat est **DERRIÈRE** la télé. **FINALEMENT**, on voit les **DEUX** chats sur le meuble, l’air **CHOQUÉ**. La télé est **TOMBÉE** par terre et l’arrière de la télé est **CASSÉ**.

### Appendix B: Example of Recall Evaluation

The left panel of the image below shows the original comic illustration, and the panel on the right shows the illustration provided by the participant. The table shows the number of points given for each gesturally marked element (**bold** indicates words that are gesturally marked elements, underline indicates the gesturally marked element being evaluated).

**Table:**

**Element**	**Points**
There are **two** birds on the **television** screen	**2**
There are **two** birds on the **television** screen	**2**
There are two **stacks** of magazines on the **floor**	**1**
There are two **stacks** of magazines on the **floor**	**2**
There is a **shelf** with a flower **vase**	**0**
There is a **shelf** with a flower **vase**	**2**

### Appendix C: Example of Discourse Comprehension Evaluation

The upper panel of the image below shows the original comic illustration, and the lower panel shows the illustration provided by the participant. The participant’s illustration demonstrates that in terms of recall, specific gesturally marked items were remembered, however, the general understanding of the narrative’s event structure is lacking. The participant drew one action (the TV breaking), yet did not include information regarding what caused the TV to break (i.e., the cats climbing on the TV, trying to catch the birds on the screen). This suggests that the participant did not understand how the cats were implicated in the narrative. The participant drew a few objects related to the story, and one action (the TV breaking), so this participant received a score of 2 for discourse comprehension.
